# Supplementary material for: Tyrosine phosphorylation of lamin A by Src promotes disassembly of nuclear lamina in interphase
Source: Life Sci Alliance. 2021 Aug 12;4(10):e202101120. doi: 10.26508/lsa.202101120 (PMC8362257; doi:10.26508/lsa.202101120)

Source Data

Figure 1

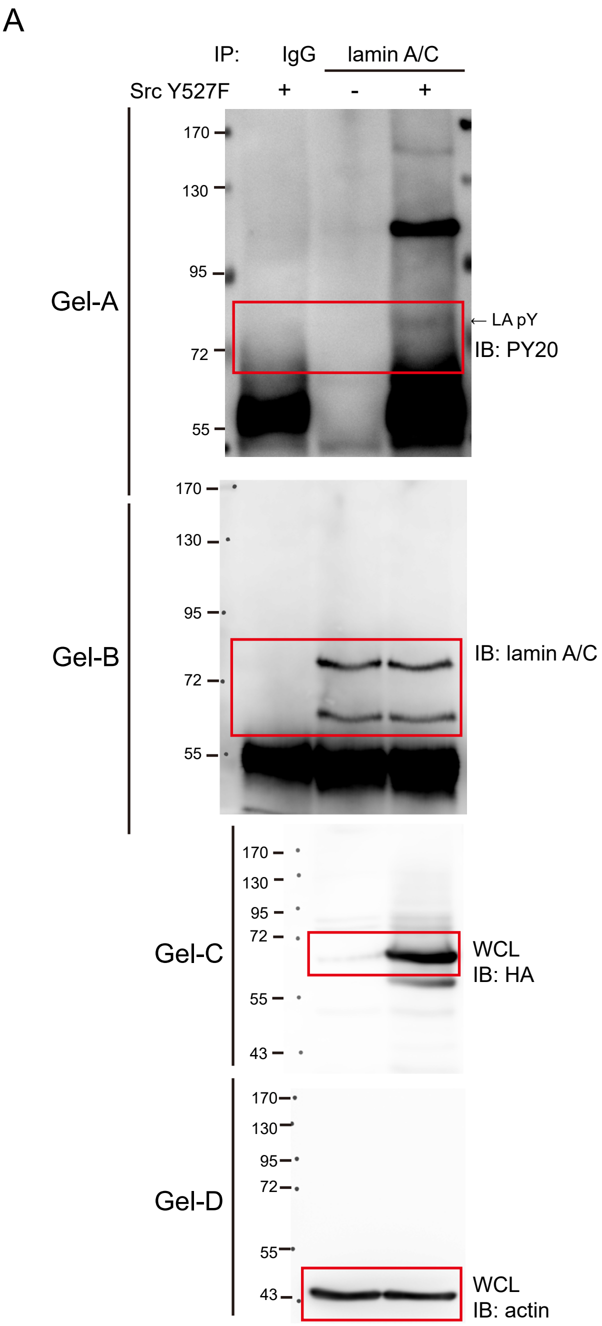

Source Data

Figure 2

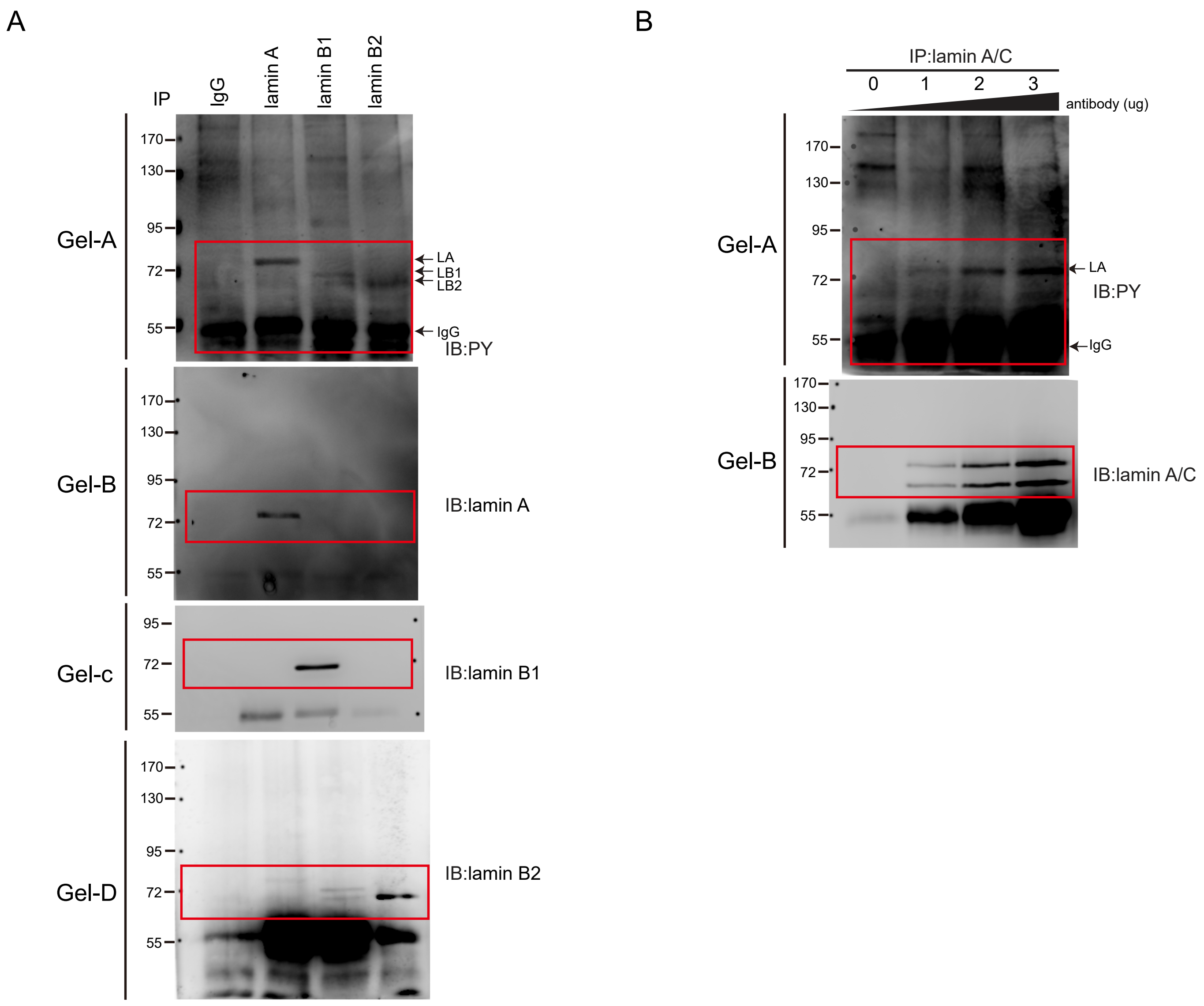

Source Data

Figure 2

C

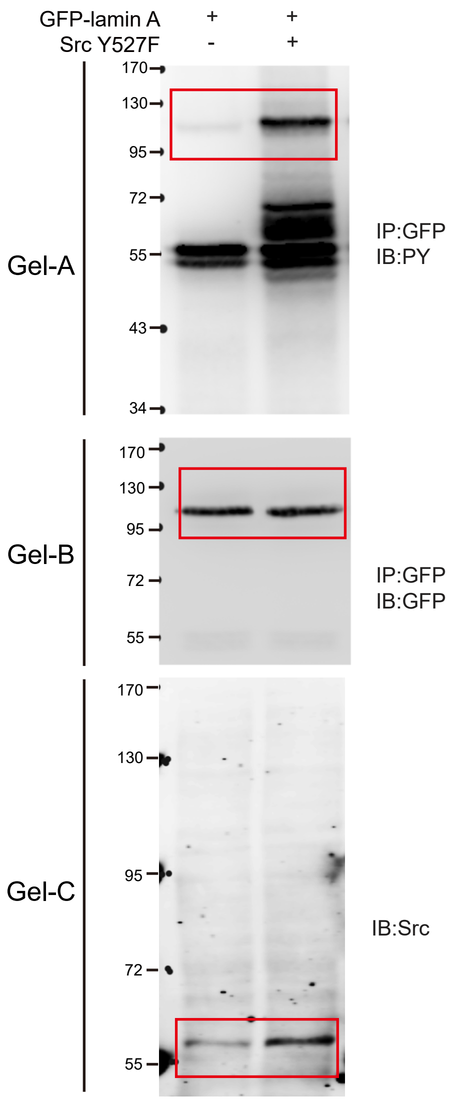

E

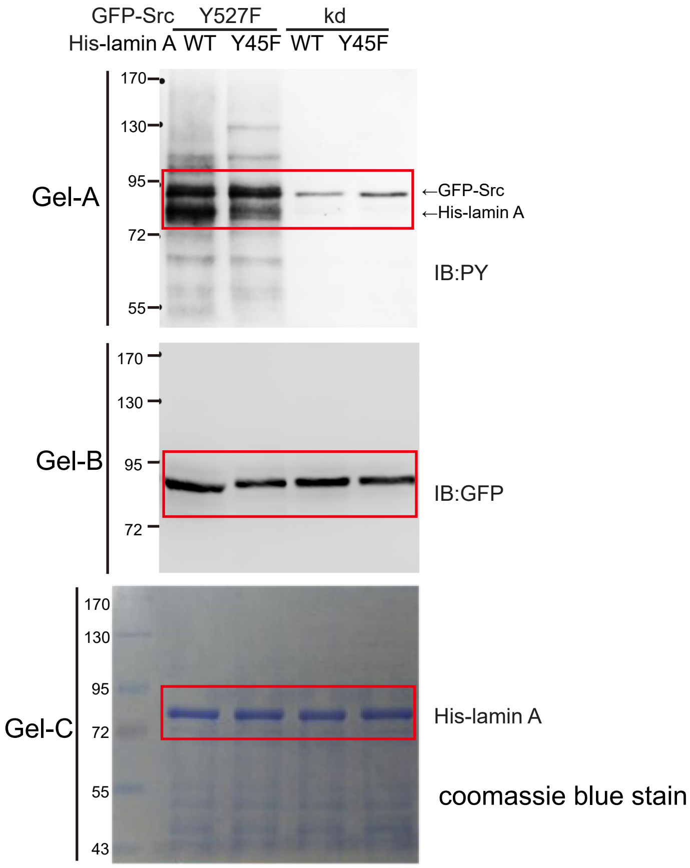

Source Data

Figure 2

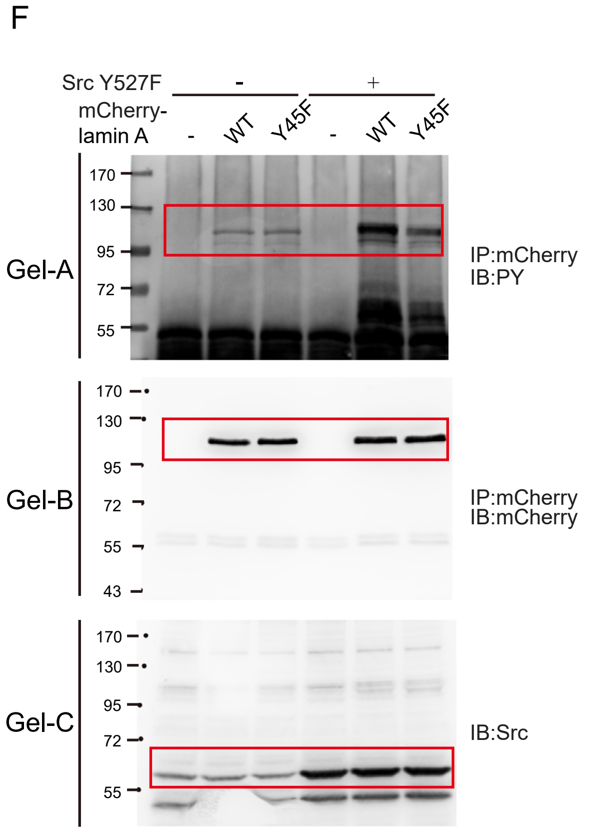

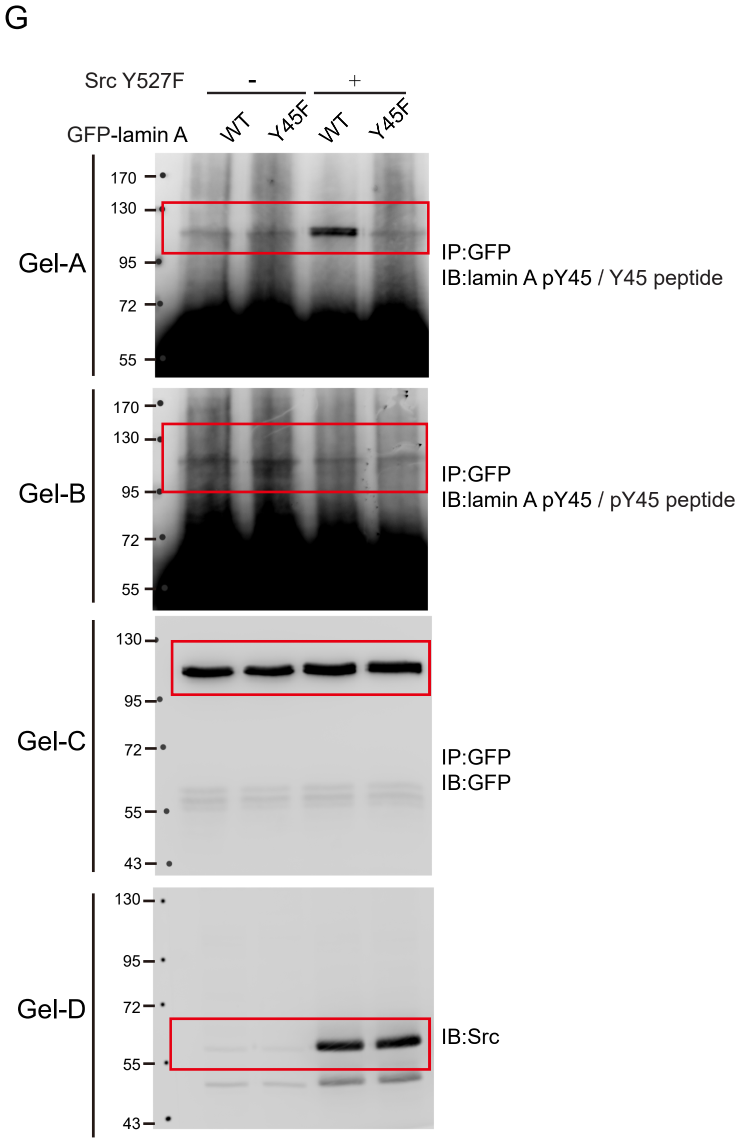

Source Data

Figure 2

H

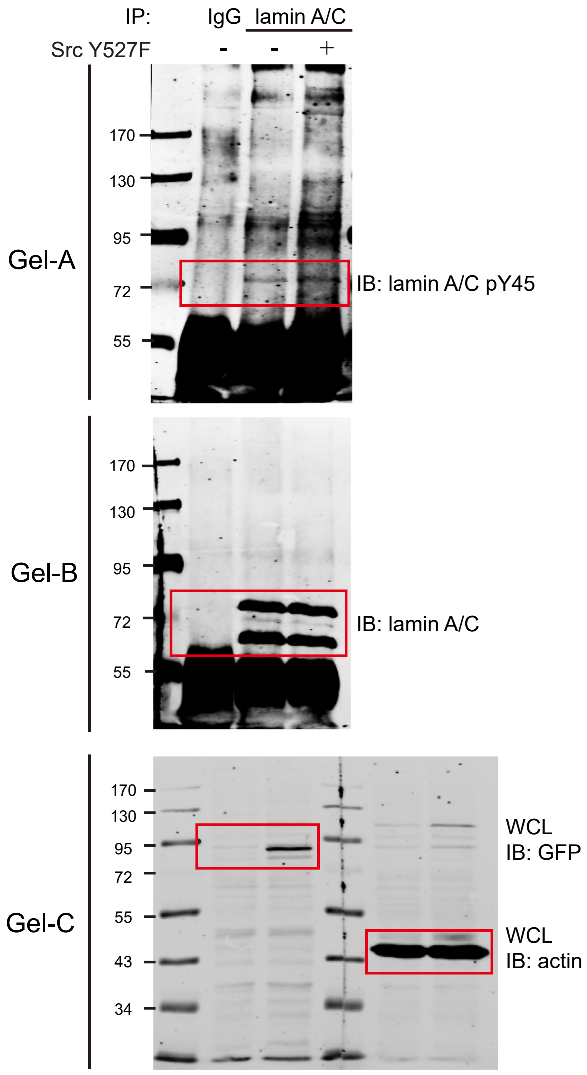

Source Data

Figure 3

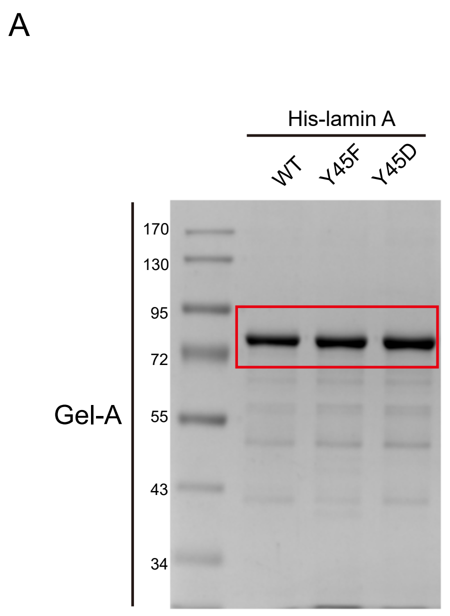

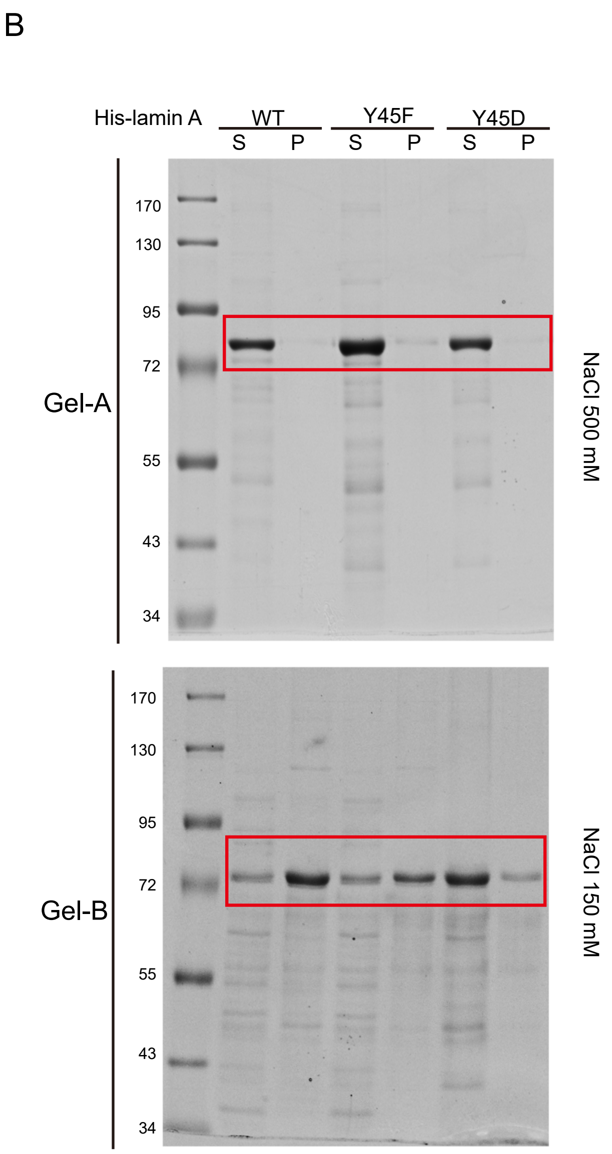

Source Data

Figure 3

C

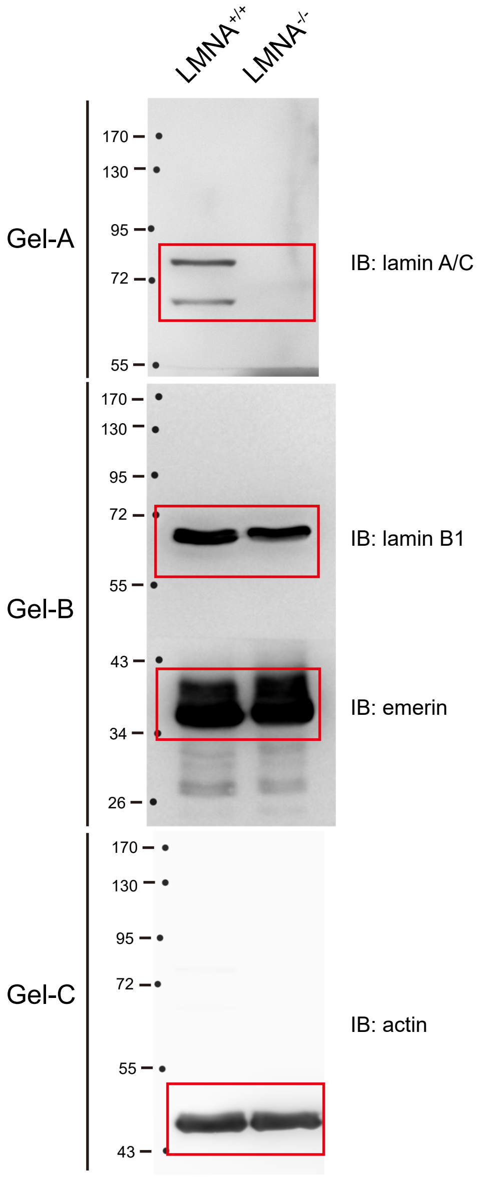

I

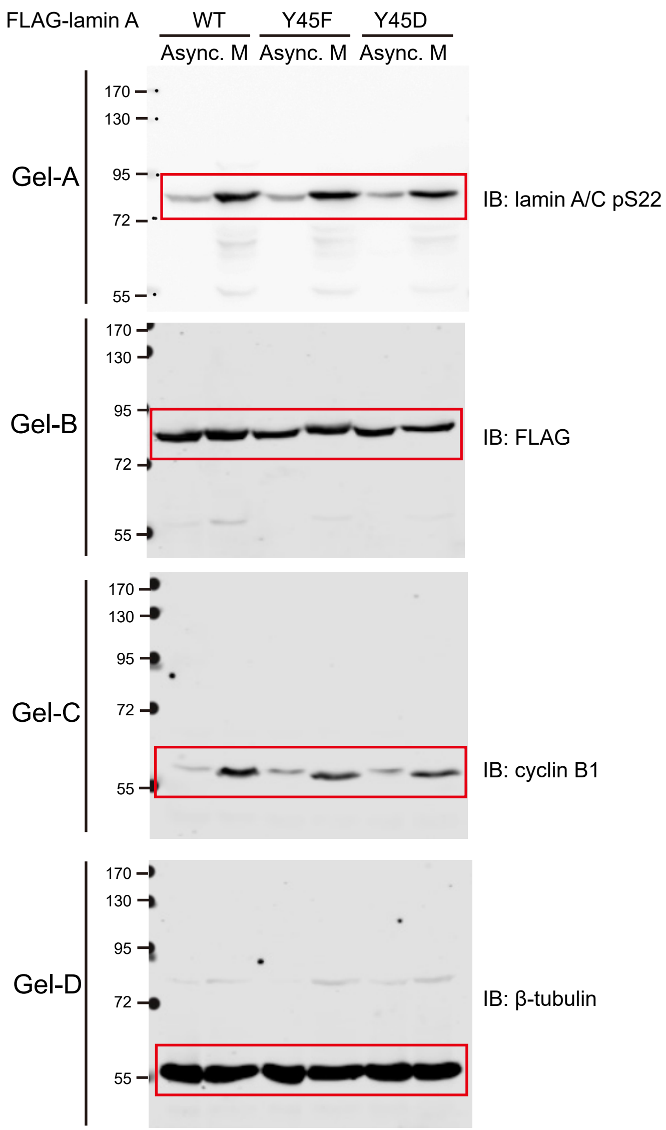

Source Data

Figure 3

J

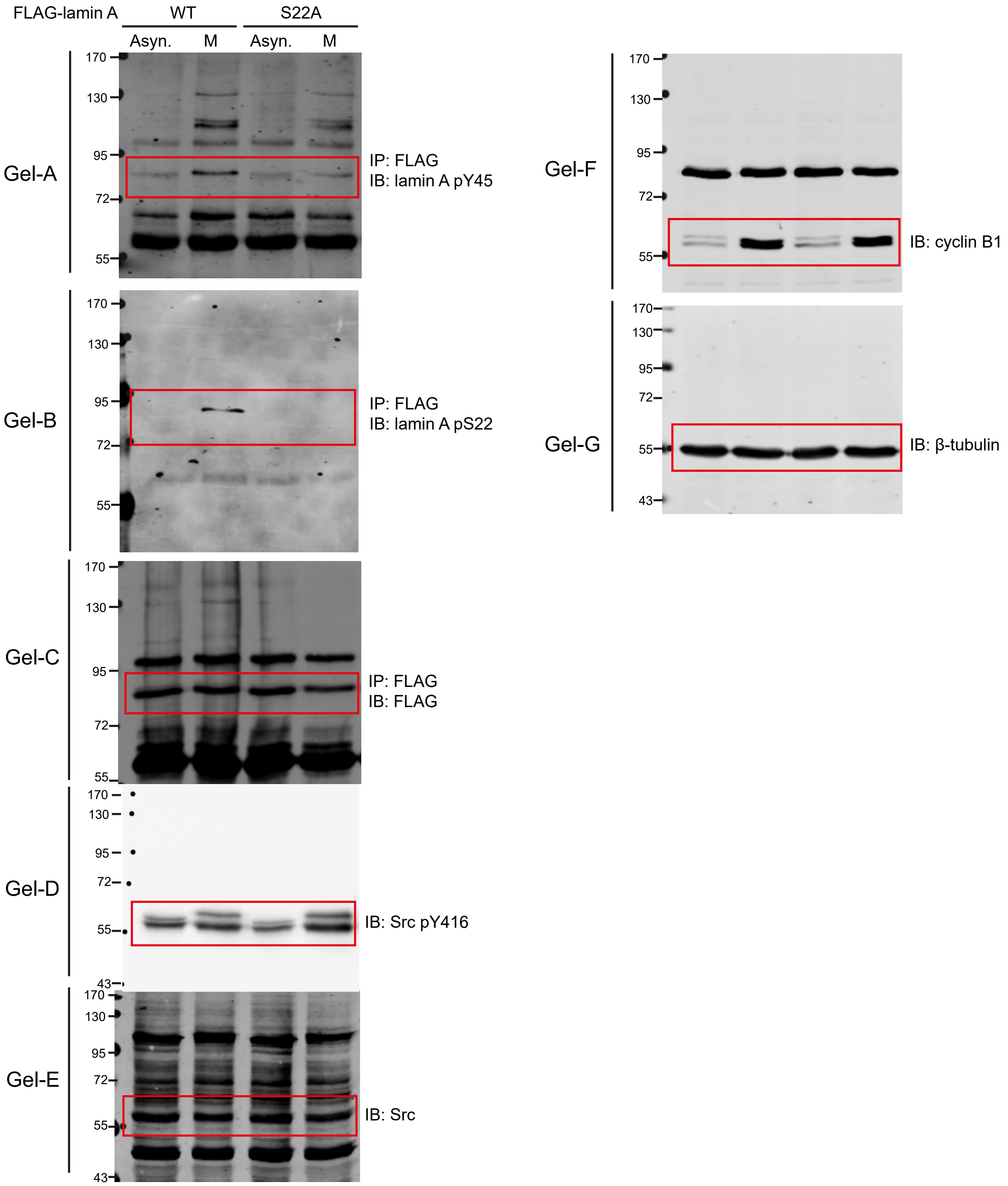

Source Data

Figure 5

A

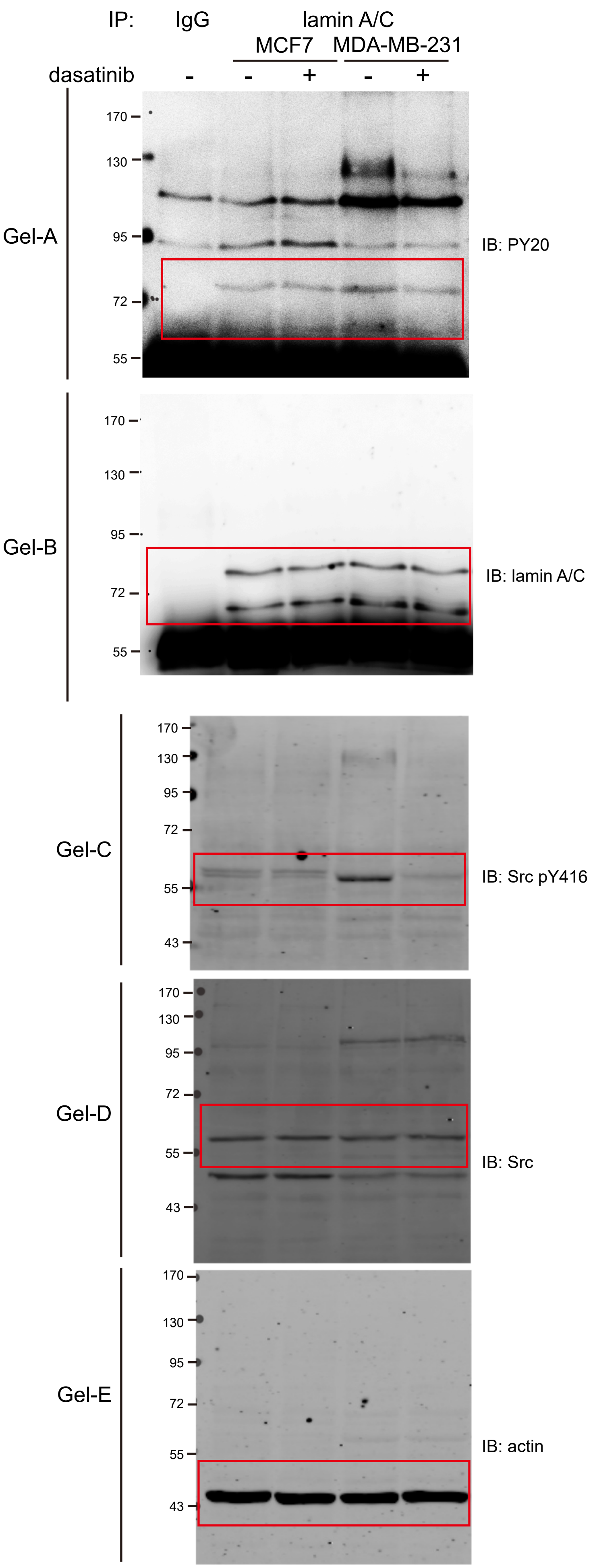

C

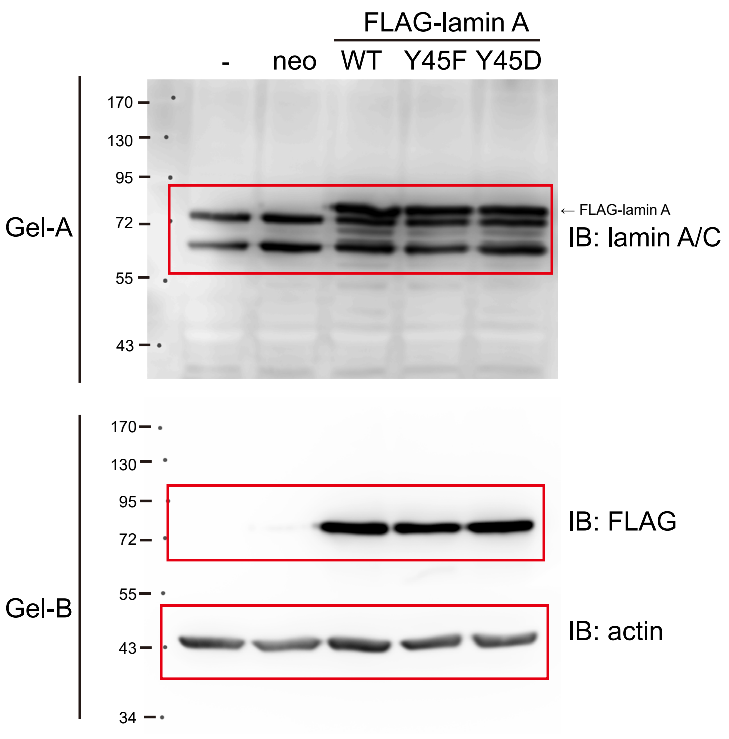

Source Data

Figure 7

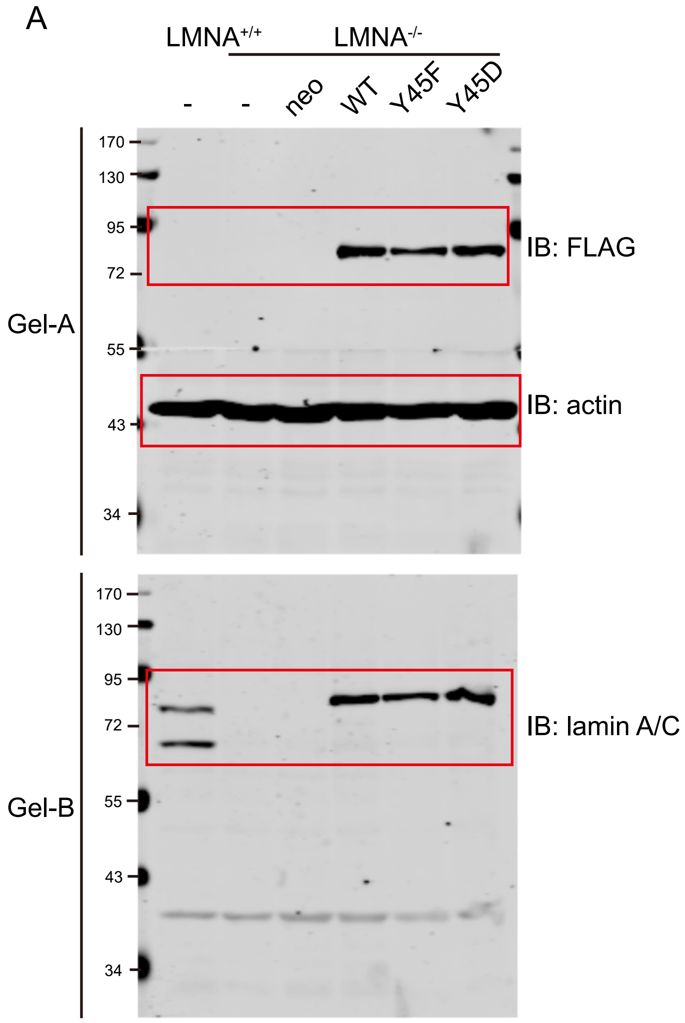

Supplement: Supplementary file 1 [file LSA-2021-01120_SdataF1_F2_F3_F5_F7.pdf]
